# Secretory co-factors in next-generation cellular therapies for cancer

**DOI:** 10.3389/fimmu.2022.907022

**Published:** 2022-08-18

**Authors:** Atsushi Okuma, Yoshihito Ishida, Taketo Kawara, Shoji Hisada, Shinsuke Araki

**Affiliations:** Center for Exploratory Research, Research and Development Group, Hitachi Ltd., Kobe, Japan

**Keywords:** CAR T cell, secretory co-factor, cytokine release syndrome (CRS), immune effector cell-associated neurotoxicity syndrome (ICANS), tumor microenvironment, solid tumor, synthetic biology

## Abstract

Since chimeric antigen receptor (CAR) T-cell therapies for hematologic malignancies were approved by the U.S. Food and Drug Administration, numerous “next-generation” CAR T cells have been developed to improve their safety, efficacy, and applicability. Although some of these novel therapeutic strategies are promising, it remains difficult to apply these therapies to solid tumors and to control adverse effects, such as cytokine release syndrome and neurotoxicity. CAR T cells are generated using highly scalable genetic engineering techniques. One of the major strategies for producing next-generation CAR T cells involves the integration of useful co-factor(s) into the artificial genetic design of the CAR gene, resulting in next-generation CAR T cells that express both CAR and the co-factor(s). Many soluble co-factors have been reported for CAR T cells and their therapeutic effects and toxicity have been tested by systemic injection; therefore, CAR T cells harnessing secretory co-factors could be close to clinical application. Here, we review the various secretory co-factors that have been reported to improve the therapeutic efficacy of CAR T cells and ameliorate adverse events. In addition, we discuss the different co-factor expression systems that have been used to optimize their beneficial effects. Altogether, we demonstrate that combining CAR T cells with secretory co-factors will lead to next-generation CAR T-cell therapies that can be used against broader types of cancers and might provide advanced tools for more complicated synthetic immunotherapies.

## Introduction

Adoptive T-cell therapies with genetic engineering to express chimeric antigen receptors (CARs) have demonstrated remarkable efficacy in patients with some B-cell malignancies and multiple myeloma (1–6). Despite successful outcomes against these specific blood tumors, CAR T-cell therapies have proven much less effective against solid tumors due to tumor heterogeneity, physical barriers preventing T-cell infiltration, and immunosuppressive tumor microenvironments (TMEs) (7). In addition, currently approved CAR T-cell therapies are associated with safety issues such as cytokine release syndrome (CRS) and immune effector cell-associated neurotoxicity syndrome (ICANS) (8). The “on-target/off-tumor” activity of CAR T cells can cause life-threatening events in some cases (9–11); therefore, it is important to develop tumor-specific CAR T-cell therapies that target novel antigens.

To overcome the issues related to current CAR T-cell therapies, numerous co-factor–expressing CAR T cells have been investigated. These co-factors can be categorized into three types based on protein localization: (i) secretory factors released by CAR T cells can affect the CAR T cells themselves as well as surrounding cells expressing a receptor for the factor; (ii) membrane proteins penetrate or associate with the CAR T-cell membrane and affect the CAR T cells and surrounding cells through ligand binding; (iii) intracellular factors such as transcription factors affect the CAR T cell itself by regulating the expression of numerous genes to dramatically change the state of the cell [e.g., Yamanaka factors: from a differentiated cell to an inducible pluripotent cell (12)]. Secretory factors are usually used to recruit other cells and/or affect cells in a wider area in a contact-independent manner, unlike membrane proteins. Moreover, secretory factors can improve the *ex vivo* expansion of CAR T cells (13), suggesting that they can be used to manufacture advanced CAR T cells. Because secretory factor genes are generally much smaller than those of membrane proteins, they can even be included in viral vectors with strict transgene size limits. In addition, the majority of candidate co-factors (cytokines and antibodies) have already been tested as anticancer agents; therefore, T cells can be genetically designed to express CAR and secretory co-factor(s) based on existing administration protocols, efficacy, and safety data.

**Figure 1:**
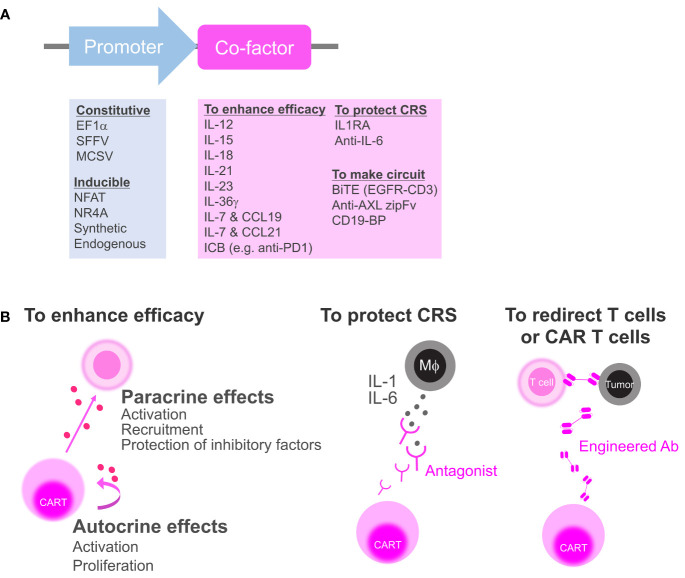
Categorization of secretory co-factors. **(A)** The list of previously reported promoters and secretory co-factors of CAR T cells (see also **Table 1**). ICB, immune checkpoint blockade; IL-1RA, IL-1 receptor antagonist; BiTE, bispecific T-cell engager; CD19-BP, CD19-containing bridging protein. **(B)** Schematics of how CAR T cell can affect the environment *via* co-factor secretion. To enhance anti-tumor efficacy, CAR T cell can secrete cytokines or ICBs to activate surrounding cells and CAR T cell itself (left). CAR T cell can secrete antagonists to block inflammatory cytokines from macrophages (Mϕ) that induce CRS (middle). CAR T can secrete bispecific antibodies or zipFvs to redirect bystander T cells or CD19-BPs to redirect CD19 CAR T cell itself (right).

In this review, we first discuss the obstacles to conventional CAR T-cell strategies and the functions that are required. Next, we provide an overview of the secretory co-factors that have already been tested in animal models or clinics from biological and clinical perspectives (**Figure 1**, **Tables 1**, **2**). Finally, we describe current knowledge of constitutive and inducible types of co-factor expression machinery which could overcome some of the issues of current CAR T-cell therapies.

**Table 1:** List of reported CAR T cells harnessing secretory co-factors.

Secretory co-factor	Promoter	CAR target	Ref

*Aim: To enhance efficacy*
IL-12	6x NFAT-RE	CEA	(14)
IL-12	LTR in RVV	CD19	(15)
IL-12	6x NFAT-RE	GPC3	(16)
IL-12	LTR in RVV	MUC16^ecto^	(17, 18)
IL-12	Endogenous CD25 promoter	CD22	(19)
IL-18	LTR in RVV	CD19	(20)
IL-18	LTR in RVV	CD19	(21)
IL-18	LTR in RVV	CD19	(22)
IL-12 or IL-18	6x NFAT-RE	GD2	(23)
IL-21	6x NFAT-RE	CD19	(24)
IL-15 & IL-21	LTR in RVV	GPC3	(25)
IL-23 (IL-12p40)	LTR in RVV	GD2, B7-H3	(26)
IL-36γ	LTR in RVV	CD19	(27)
IL-15	LTR in RVV	CLL-1	(28)
IL-15	LTR in RVV	GD2	(29)
IL-15	LTR in RVV	IL-13Rα2	(30)
CD40 agonist	LTR in RVV	MSLN	(31)
IL-7 & CCL19	LTR in RVV	CD20	(32)
IL-7 & CCL19	LTR in RVV	GPC3, MSLN	(33)
IL-7 & CCL21	LTR in RVV	CLND18.2	(34)
Anti–PD-1 scFv (E27)	LTR in RVV	CD19	(35)
Anti–PD-1 scFv (E30)	LTR in RVV	EGFR	(36)
Anti–PD-1 scFv	LTR in RVV	CD19	(37)
Anti–PD-1–TGF-βRII ectodomain	LTR in RVV	CD19	(38)
Anti-CD47, Anti–PD-L1, Anti-CTLA4	LTR in RVV, CMV	PD-L1, EIIIB	(39)
Anti–PD-L1 scFv	LTR in RVV	CAIX	(40)
***Aim: To protect CRS***			
IL-1RA	LTR in RVV	CD19	(41)
Anti–IL-6 and IL-1RA	LTR in RVV	CD19	(42)
Anti–IL-6 scFv and IL-1RA	LTR in RVV	CD19, BCMA	(43)
***Aim: To make circuit***			
BiTE EGFR-CD3	EF-1α	EGFRvIII	(44)
Various factors	Gal4-UAS (SynNotch)	CD19	(45)
Anti-AXL zipFv	4x NFAT-RE	HER2	(46)
CD19–anti-Her2 bridge protein	CMV	CD19	(47)
CD19–anti-CLEC12A bridge protein	MSCV	CD19	(48)

LTR, long terminal repeat; RVV, retroviral vector; NFAT-RE, nuclear factor of activated T cell response element; BiTE, bispecific T-cell engaging antibody; UAS, upstream activation sequence.

**Table 2:** Clinical trials of immune checkpoint blockade-expressing CAR T cells.

ICBs	CAR target	Sponsor	Phase	ClinicalTrials.gov ID	Report
Anti–PD-1 Anti-CTLA4	MUC1	Shanghai Cell Therapy Research Institute	1/2	NCT03179007	
Anti–PD-1 Anti-CTLA4	EGFR	Shanghai Cell Therapy Research Institute	1/2	NCT03182816	(49)
Anti–PD-1 Anti-CTLA4	MSLN	Shanghai Cell Therapy Research Institute	1/2	NCT03182803	
Anti–PD-1	MSLN	Ningbo Cancer Hospital	1/2	NCT03030001	
Anti–PD-1	EGFR	Ningbo Cancer Hospital	1/2	NCT02873390	
Anti–PD-1	EGFR	Shanghai International Medical Center	1/2	NCT02862028	
Anti–PD-1 Anti–PD-L1	EGFRvIII	Shenzhen Geno-Immune Medical Institute	1/2	NCT03170141	

## Challenges of current CAR T-cell therapies

### Adverse events: CRS and ICANS

The clinical success of CD19-directed CAR T cells has also been accompanied by various limitations. CRS, the most common adverse event of CAR T-cell therapies, is caused by the overproduction of proinflammatory cytokines and mainly correlates with tumor burden (8). It has recently been suggested that during CRS, CD40L on CAR T cells and factor(s) from dead cells known as “danger signals” stimulate monocytes/macrophages to release interleukin-1 (IL-1) (41, 50). In addition, granulocyte-macrophage colony-stimulating factor (GM-CSF) from CAR T cells stimulates monocytes/macrophages to simultaneously proliferate at the inflammatory site (41, 43, 51). After IL-1 overproduction, the monocytes/macrophages produce IL-6, which plays a pivotal role in CRS (50). Current clinical protocols to treat CRS include glucocorticoids and/or IL-6 blockade (tocilizumab) (1, 52), whereas preemptive or early intervention with tocilizumab has been reported to prevent severe CRS (53, 54). IL-1 blockade (anakinra) is another promising strategy that is currently in clinical trials (55). ICANS is a severe and life-threatening adverse effect of CAR T-cell therapy (56, 57); however, the induction mechanisms remain unclear and few working therapeutic protocols have been verified. Although ICANS is associated with early systemic inflammation and CRS, the rate of which can be decreased through early intervention with tocilizumab, this therapeutic strategy does not affect the frequency of severe ICANS (58, 59). Conversely, prophylactic or early intervention with high-dose anakinra has yielded promising results against ICANS (55, 60). Together, these findings suggest that constitutive IL-6 and IL-1 blockade during CAR T-cell therapy may prevent CRS and ICANS, respectively.

### Antigen specificity: “On-target, off-tumor” effects

Another limitation of CAR T-cell therapy is specificity. As of March 2022, four of the six CAR T-cell therapies approved by the U.S. Food and Drug Administration target CD19 as a tumor marker (1–4), whereas the others target B-cell maturation antigen (BCMA) (5, 6). To expand their application, it is necessary to develop CAR T-cell therapies targeting new antigens; however, a lack of specificity can lead to “on-target, off-tumor” effects which cause life-threatening toxicity depending on the non-pathogenic cell type(s) that express the target antigen. For instance, a patient who received CAR T cells targeting the tumor antigen human epidermal growth factor receptor 2 (HER2) experienced rapid respiratory failure, multi-organ dysfunction, and subsequent death due to reactivity against pulmonary epithelia with slight HER2 expression (10). This issue could be solved by CAR T-cell strategies that can clearly discriminate between cancer cells and normal cells based on an antigen density threshold, since tumor-associated antigens are expressed at much higher levels in tumors than in normal tissues (61). In addition, *AND* logic could be applied to produce (A *AND* B) CAR T cells that can recognize cells expressing both antigens A and B, but not cells expressing only A or B (46, 62, 63). Even if neither antigen is specific to the tumor, the simultaneous expression of both antigens could be tumor-specific and thus limit “on-target, off-tumor” effects.

### Efficacy against solid tumors

The other major limitation of CAR T-cell therapies is their ability to fight solid tumors, which is reduced by intratumor heterogeneity, an immunosuppressive TME, and/or physical barriers (7). Intratumor heterogeneity makes it difficult to identify appropriate tumor-specific antigens. Although non-engineered T cells can be primed to react to neoantigens or tumor-associated antigens, they are usually suppressed by components of the TME, such as regulatory T (T_reg_) cells and immune checkpoint ligands. Because the TME also interferes with CAR T-cell activity, numerous studies have attempted to modify the interaction between CAR T cells and the TME. For instance, programmed cell death protein 1 (PD-1) knockout CAR T cells can avoid PD-1–PD-L1 (programmed death-ligand 1) immune checkpoint signals (64), whereas dominant-negative transforming growth factor–β (TGF-β) receptor–expressing CAR T cells can attenuate T_reg_ maintenance by blocking TGF-β (65). The TME also has much lower levels of homeostatic T-cell–supportive cytokines than lymphoid tissues, which may explain the limited persistence of tumor-infiltrating CAR T cells and tumor-reacting T cells. Indeed, CAR T cells that produce IL-7 and CCL19 to mimic T-zone function in lymphoid tissues have been reported to exhibit strong efficacy in animal solid tumor models, mastocytoma cell line–derived xenografts (CDXs), hepatocellular carcinoma (HCC) patient-derived xenografts (PDXs), and pancreatic carcinoma CDXs (32, 33). Prior to their activation, intratumor CAR T cells and tumor-reacting T cells must penetrate and survive in tumors; however, the stromal structure of solid tumors acts as a physical barrier to protect against T-cell infiltration. CAR T cells harnessing the extracellular matrix-degrading enzyme heparanase are expected to improve tumor infiltration (66).

## Secretory co-factors for enhancing CAR T-cell efficacy

### IL-12

IL-12 is a proinflammatory cytokine that has been repeatedly reported as a co-factor for CAR T cells. IL-12 induces the differentiation of CD4^+^ T cells into a helper T-cell (T_H_1) subtype that release interferon-γ (IFN-γ) and support the cytotoxic activity of CD8^+^ T cells. Consequently, IL-12 was expected to improve CAR T-cell cytotoxicity; however, IL-12–expressing CAR T cells have been reported to enhance monocyte (23) and T cell (16) recruitment, macrophage antigen presentation (14), and CAR T-cell persistence (19) rather than their cytotoxicity. Local recombinant IL-12 delivery can reshape the immunosuppressive TME (67, 68) and several clinical trials for tumor therapies with recombinant IL-12 administration have been conducted in recent decades (69). Unfortunately, high-dose systemic IL-12 treatment can cause life-threatening adverse events (70, 71) and milder regimens had no effect on advanced renal cell cancer as they delivered insufficient local concentrations of IL-12 to the TME (71). CAR T cells can carry IL-12 into tumors and IL-12 expression systems with inducible promoters, such as nuclear factor of activated T cell (NFAT) promoter, may safely minimize systemic IL-12 leakage (14, 16, 23). Because IL-12 is the most clinically characterized CAR T-cell co-factor, it could reasonably be used in future CAR T-cell therapies.

### IL-15

IL-15 stimulates T cells and NK cells to enhance their proliferation and cytotoxic capacity. The administration of recombinant IL-15 has been reported to accelerate the anti-tumor activity of cytotoxic T cells in mice (72) and recent reports have shown that IL-15 can polarize T cells to central memory and stem cell memory subtypes rather than the effector subtype and thereby prevent the upregulation of inhibitory receptors associated with T-cell exhaustion during *ex vivo* expansion (73–75). The anti-tumor activity of IL-15–expressing CAR T cells (28–30) is mainly thought to derive from cell-autonomous effects and their effects on locally colonized non-engineered T cells. However, clinical trials have indicated that systemic recombinant IL-15 administration to treat metastatic cancers can result in hypotension, thrombocytopenia, and liver toxicity (76). Consequently, it has been reported that even IL-15–expressing CAR T cells have lethal toxicity in an acute myeloid leukemia CDX model and TNF-α blockade ameliorated the toxicity of IL-15 (28). Thus, inducible IL-15 production system like NFAT–IL-12 would be tried to improve safety to avoid high systemic IL-15 levels.

### IL-18

Like IL-12, IL-18 activates T_H_1 and NK cells to proliferate and release IFN-γ (20). In addition, IL-18–expressing CAR T cells recruit and activate endogenous anti-tumor immune cells in the TME (22). A previous animal study has suggested that IL-18 expressed by CAR T cells are more effective against advanced pancreatic tumors than IL-12 (21). Although systemic IL-18 administration has been reported to exert moderate adverse effects in clinical trials (77), IL-18 could be a safer and more effective co-factor than IL-12. Combination therapy with CAR T cells and recombinant IL-18 would be more costly than monotherapy with IL-18–producing CAR T cells; however, the additional clinical benefits of IL-18–producing CAR T cells compared to the combination therapy, such as specific efficacy and reduced adverse events, must be explored.

### IL-21

IL-21 is a cytokine derived from follicular helper T cells that promotes high-affinity immunoglobulin production by B cells, T_H_1 and T_H_17 differentiation, and CD8 T-cell proliferation (78). Recombinant IL-21 supplementation has been reported to maintain an early memory T subtype during *ex vivo* CAR T-cell expansion (24). Although CAR T cells with activation-dependent IL-21 secretion (NFAT promoter-IL-21) displayed increased tumor infiltration in a chronic lymphocytic leukemia CDX model, no obvious improvement in anti-tumor efficacy has been reported (24). However, CAR T cells with constitutive expression of both IL-15 and IL-21 improved potency in a HCC CDX (25). To proceed to the clinical application, IL-21 needs further study to explore the optimal cytokine combination.

### IL-23

IL-23 has recently been reported as a promising secretory co-factor for CAR T cells (26). IL-23 is composed of two subunits; p40 (shared with IL-12) and p19. In activated T cells, the IL-23 receptor and p19 subunit are upregulated, but not the p40 subunit; therefore, Ma et al. engineered CAR T cells to express p40 to compensate for the cell-autonomous IL-23–IL-23R axis (26). These p40-expressing CAR T cells not only had a better safety profile but also displayed better efficacy against neuroblastoma and pancreatic cancer CDXs by promoting antigen-dependent proliferation and CAR T-cell persistence compared to IL-15 or IL-18. The data of this head-to-head study are valuable, and IL-23 should be tested with various types of CAR T cells to prove the concrete superiority of IL-23.

### IL-36γ

IL-36 is a member of the IL-1 superfamily, like IL-18, that stimulates the NF-κB/AP-1 signaling pathway. The IL-36 receptor complex, which is composed of IL-36R (also known as IL-1RL2) and IL-1RAP, is expressed on epithelial cells, myeloid cells, and T cells. In terms of tumor immunity, IL-36 can induce anti-tumor immune responses, including the activation of T_H_1 (79), CD8^+^ T, γδT, and NK cells (80). In a recent study, IL-36γ–producing CAR T cells exerted superior therapeutic efficacy in leukemia xenograft and allograft mouse models through CAR T-cell self-activation and antigen-presenting cell activation (27); however, their effects against solid tumors have not yet been reported.

### IL-7 and CCL19 or CCL21

IL-7 and CCL19 secreted from T-zone fibroblastic reticular cells recruit endogenous immune cells such as T cells and dendritic cells (DCs) from the periphery. To fight heterogeneous tumor cells, it is considered to be important to make the TME “hot” by recruiting endogenous immune cells; therefore, IL-7 and CCL19 have been combined as secretory co-factors for CAR T cells in a mastocytoma model (32). IL-7– and CCL19-expressing (7 × 19) CAR T cells showed a stronger therapeutic effect against mouse mastocytoma with increased endogenous DC and T-cell infiltration (32). A clinical trial of advanced carcinoma with glypican-3 or mesothelin expression found that two of the six patients had a complete or partial response to 7 × 19 CAR T-cell treatment without CRS or ICANS (33). Most recently, IL-7– and CCL21-expressing (7 × 21) CAR T cells were reported to yield better efficacy than 7 × 19 CAR T cells in mouse solid tumor models of pancreatic carcinoma, breast cancer, and HCC without preconditional lymphodepletion (34). Importantly, more DCs and T cells and fewer blood vessels were observed at the tumor sites of mice treated with 7 × 21 CAR T cells. CCL21 shares the same receptor (CCR7) with CCL19; however, the differential ability of CCL19 and CCL21 for desensitizing CCR7 (81) and/or the ability of CCL21 for binding other receptors like CXCR3 might cause the differential anti-tumor potency.

### Anti–PD-1/PD-L1

The major immune inhibitory receptor PD-1 and its ligand PD-L1 are molecular targets of immune checkpoint blockade (ICB) therapies for various tumors (82–84); however, the therapeutic efficacy of these therapies depends on the TME immune status and the frequency of somatic mutations/neoantigens in tumor cells (83, 85, 86). CAR T cells could be an ideal booster to expand the applications of PD-1/PD-L1 ICB as they can recognize non-mutated proteins rather than neoantigens and can trigger endogenous immune reactions against tumors. In addition, ICBs could improve the persistence and efficacy of CAR T cells by altering the immunosuppressive TME (86–88). Clinical investigations of therapies combining CAR T cells and systemic ICBs are currently ongoing (89) and CAR T cells that secrete PD-1/PD-L1 blockades are also in development (35–40). Indeed, CAR T cells secreting PD-1 blockades have shown better results in a mouse model of pulmonary mucoepidermoid carcinoma than CAR T cells alone or CAR T cells combined with systemic PD-1 blockade (37). Several PD-1/PD-L1 blockade-secreting CAR T cells have been developed and are currently in clinical trials (**Table 2**) (90). Other approved ICBs against the inhibitory receptors CTLA-4 (84) and LAG-3 (91) and the upcoming ICB-targeting CD47, which inhibits phagocytosis-mediated cancer cell removal (92–94), may be also good CAR T-cell co-factors. Thus, the optimal co-factors for each cancer could be selected based on existing evidence from ICB monotherapies.

### IL-1 and/or IL-6 blockade to protect against CRS and ICANS

Treatment with the anti–IL-6 drug, tocilizumab, can prevent CRS in mouse models but not abolish neurotoxicity (41, 50), whereas the natural IL-1 receptor antagonist (IL-1RA; human IL-1RA also known as anakinra) can prevent severe CRS and fatal neurotoxicity. To maximize the preventative effects of IL-1 blockade, mouse IL-1RA has been utilized as a secretory co-factor for CAR T cells that reduced CRS-related mortality without decreasing anti-tumor activity in a mouse model of CRS (41). Recent early-stage clinical investigations of CAR T cells that autonomously secrete anti–IL-6 and IL-1RA resulted in moderate CRS with neurotoxicity during CRS (**Figure 1B** **middle**) (42, 43). Considering the promising clinical effects of preemptive tocilizumab or anakinra administration (53–55, 58–60), CAR T cells incorporating those secretory co-factors may be the closest to practical application.

## Engineered antibodies to redirect bystander T cells or CAR T cells

Bispecific T-cell engager (BiTE) is a tool that can be used to redirect T cells to attack tumor cells by acting as a bridge between CD3 and a target antigen (**Figure 1B** **right**). To achieve both tumor specificity and overcome tumor heterogeneity, EGFRvIII CAR T cells expressing BiTE against EGFR were developed against glioblastoma (44). EGFRvIII is a glioblastoma-specific tumor antigen and EGFR is highly expressed in glioblastoma and normal tissues, such as skin, but not in normal brains. EGFRvIII CAR acted as an anchor to the tumor, whereas EGFR-targeted BiTE redirected both CAR T cells and bystander T cells to attack the heterogeneous tumor. In addition, bicistronic CAR T cells have demonstrated potent and specific efficacy against heterogeneous tumors in animal models of glioblastoma (44).

**Figure 2:**
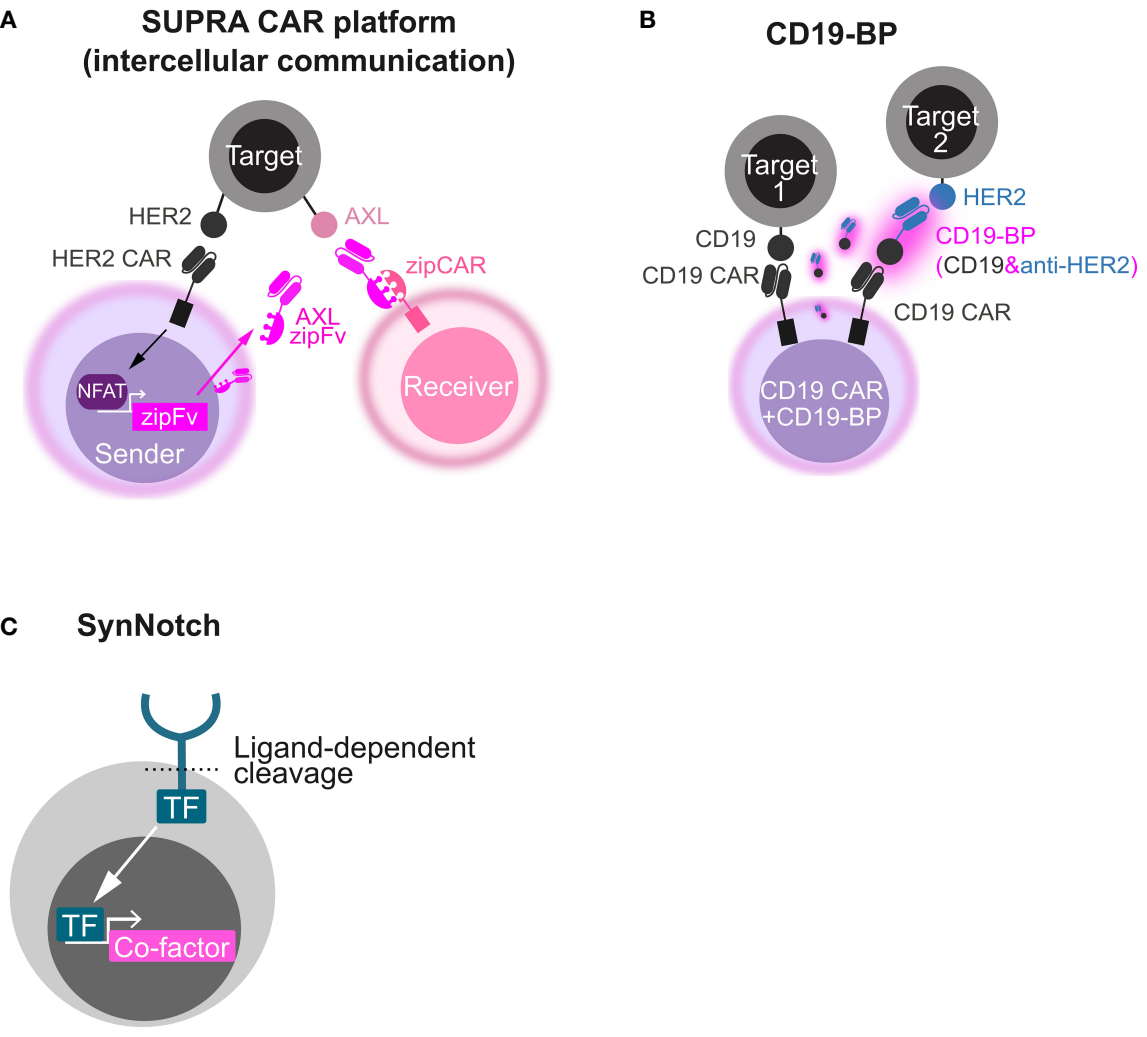
Synthetic biology tools to make circuits by secretory co-factors. **(A)** Schematics of intercellular *AND* gate with the SUPRA CAR platform. A HER2 CAR-expressing sender cell secretes AXL zipFv in a CAR stimulus–dependent manner using the NFAT promoter. When AXL zipFv bridges a zipCAR-expressing receiver cell and AXL on a target cell, the receiver cell is activated. **(B)** Schematics of *OR* gate with a CD19 bridge protein (CD19-BP). This engineered cell expresses both CD19 CAR and the CD19-BP that is composed of recombinant CD19 and anti-HER2 scFv. Secreted CD19-BP engages CD19 CAR and a HER2-expressing target cell. **(C)** Schematics of how synNotch induces a co-factor. Upon ligand recognition by the synNotch receptor, a transcription factor (TF) integrated in the synNotch cytoplasmic domain is cleaved and released. The released TF induces the expression of a custom co-factor.

This strategy can be applied to strict intercellular circuits by changing the co-factor and its expression machinery. For instance, in SUPRA platform, the CAR stimulus-inducible NFAT promoter was utilized to express an engineered antibody, AXL zipFv, that acts as a bridge between T cells expressing the compatible chimeric receptor zip CAR and the tumor antigen AXL (46). An intercellular *AND* gate (inputs, HER2 and AXL; output, receiver cell activation) was developed using sender cells harnessing HER2 CAR and the NFAT-AXL zipFv cassette and receiver cells harnessing the compatible zipCAR that were co-cultured with HER2 and AXL double-positive cells (**Figure 2A**). Beyond conventional CAR T-cell therapies, these synthetic biology techniques can engineer beneficial cell-cell communication *via* artificial secretory factors.

Another strategy utilizing recombinant CD19-containing bridging proteins (CD19-BPs) can redirect CD19 CAR T cell itself (**Figure 2B**) (47, 48). CD19 CAR T cells that secrete CD19-BP–targeting HER2 killed HER2-positive cells and CD19-positive cells (*OR* gate) and showed the comparable efficacy to HER2 CAR T cells in an ectopic HER2-positive ovarian cancer CDX model (47). In AML CDX models, CD19 CAR T cells that secrete CD19-BP–targeting CLEC12A exhibited the similar anti-tumor activity to CLEC12A-targeting CAR T cells (48). Like the BiTE secretion system, this system might be useful to tackle tumor heterogeneity and relapse due to antigen escape. CD19-BPs might have stronger effect than BiTE in that CAR signal is leveraged, although CAR T-cell exhaustion caused by repeated stimulation would be a concern in this system.

## Co-factor expression machineries

### Constitutive expression

Utilizing a strong constitutively active promoter can be the best way to maximize the expression of secretory co-factors. Compared to systemic administration, the area of co-factor efficacy can be regulated by CAR T-cell localization, even if they produce the co-factor at very high levels, thereby reducing the side effects of the co-factor. Despite the remarkable anti-tumor efficacy of IL-12 in various animal models, clinical trials of recombinant IL-12 showed severe toxicity, including mortality (70, 95). Therefore, various localized IL-12 delivery strategies that could be more effective and less toxic, including IL-12–expressing CAR T cells (15, 17, 18), are currently in clinical trials (96).

Since retrovirus vectors are generally used for T-cell transduction, the expression of secretory co-factors and CAR is often driven by the retroviral long terminal repeat (LTR) promoter or the CMV (cytomegalovirus), EF1α (elongation factor 1α), PGK (phosphoglycerate kinase), and SFFV (spleen focus-forming virus) promoters (97–100). In many cases, the EF1α and SFFV promoters are stronger, but do not always lead to a better transcriptional activity. In addition, CAR overexpression can lead to tonic signals and premature exhaustion (100, 101). Various gene drivers can be constructed by combining promoters, introns, and enhancers and can be optimized for the application of interest.

### NFAT promoters

To avoid the unexpected effects of co-factors, inducible CAR/TCR activation–inducible promoters with low background activity have been used to localize co-factor delivery. NFAT promoters including NFAT response elements (REs) are widely used to monitor TCR activation. For instance, an NFAT promoter driving IL-12 secretion from *ex vivo* expanded tumor-infiltrating lymphocytes (TIL) was tested for metastatic melanoma clinical therapy (102). Clinical toxicity was still observed after high-dose infusion, possibly due to non-localized TILs with unknown TCR stimulation. Combining NFAT promoters with CARs could allow better control over the input signal to reduce unexpected co-factor expression. IL-12 and IL-18 have been selected as co-factors to be induced by NFAT promoters (14, 16, 23) due to their systemic toxicity, which can cause fever, leukopenia, and on-study death (20, 70, 71). However, there is concern that the NFAT promoter might not be strong enough to express less toxic co-factors, such as PD-1 blockades and anti–IL-6. Increasing the number of NFAT-RE repeats (103) and changing the minimal promoter (23) have been reported to improve the NFAT promoter, resulting in stronger induction of the fluorescent reporter EGFP. Thus, customized NFAT promoters could have various applications.

Inducible constructs, such as the CAR and NFAT co-factor system, tend to involve the loading of a larger fragment that contains two promoter-coding gene cassettes: a constitutively active promoter, CAR, an inducible promoter, and a co-factor. Transposon systems can insert a much larger DNA fragment into the genome than viral vectors with a strict transgene size (104). Indeed, the transposon system piggyBac has been used to engineer CAR T cells harnessing NFAT–IL-21 instead of viral vectors (24). Large gene transfer techniques such as these could therefore be used to improve gene therapies by allowing them to become more complex and contain multiple gene cassettes.

### NR4A promoter

The CAR/TCR-inducible NR4A promoter has been reported to have a comparable maximum activity but greater sensitivity than the conventional NFAT promoter (105). CAR T cells with the NR4A promoter showed greater responses when they met cancer cells with low target antigen expression. In addition, the NR4A promoter improved poorly responsive CAR T cells by inducing the higher expression of T-cell–supportive cytokines. Therefore, the NR4A promoter could expand the applications of co-factor–harnessing CAR T cells by improving the co-factor expression in non-ideal situations such as CAR T cell against a weakly immunogenic target and poorly responsive CAR T cells derived from chemotherapy-received patients.

### Endogenous promoters

Genome-editing technologies have enabled us to generate CAR T cells in which an endogenous promoter drives CAR expression. Utilizing well-characterized endogenous promoters can not only strictly regulate CAR expression but also that of multiple additional transgenes. CAR is usually inserted in the *TRAC* locus, which encodes TCRα. This produces uniform and cell type–specific CAR expression (19, 106, 107) that enhances CAR T-cell potency without unexpected differentiation and exhaustion. Sachdeva et al. reported CAR T cells producing IL-12 under the control of *CD25* or *PDCD1* regulatory elements (19), suggesting that various promoter types can be applied to express co-factors. Therefore, endogenous promoters and highly efficient techniques for gene transfer and transgene genome integration should be validated.

### Other synthetic promoters

Hypoxia-inducible promoters are often used for hypoxic TME-specific CAR expression and are composed of hypoxia-responsive elements that allow HIF1α-dependent transcription under low oxygen conditions (108, 109). To recognize the TME through both hypoxia and inflammation, synthetic promoters composed of multiple consensus promoter response element sequences for IFN-γ, TNF-α, and hypoxia were tested in human T cells (110). *In vitro* experiments confirmed additive gene expression due to IFN-γ, TNF-α, and hypoxia; thus, synthetic promoters could provide CAR T cells with artificial genetic circuits that can allow more complex therapeutic applications.

### SynNotch

The SynNotch system is a type of molecular switch for artificial gene circuits that is composed of an extracellular antigen recognition domain (single-chain variable fragment, scFv), a Notch core regulatory region, an engineered transcriptional factor as an intracellular domain, and a gene expression cassette with a promoter that is compatible with the transcription factor (111). Once scFv recognizes the antigen on target cells, the Notch core is cleaved by a metalloprotease and γ-secretase. The transcription factor is released and translocated to the nucleus, triggering the transcriptional activation of the gene of interest (**Figure 2C**). Roybal et al. developed T cells to express CAR after synNotch receptor activation as a combinatorial antigen-sensing system (*AND* gate) (63). The synNotch system was subsequently modified so that the engineered cells could secrete diverse therapeutic factors in an antigen-specific manner (45, 112). Because synNotch itself does not induce CAR/TCR activation, the system could be used for the local delivery of biologics rather than amplifying CAR T-cell efficacy. In addition, synNotch allows stronger gene induction and more precise control of signal pathways than other inducible promoters like the NFAT promoter. An artificial transcription factor consisting of a Gal4 DNA binding domain fused to a tetrameric VP64 viral transcriptional activator domain (Gal4-VP64) is often used and has great transcriptional activity. Gal4-VP64 specifically binds to an upstream activation sequence (UAS) and induces downstream gene expression. Artificial transcriptional factors like Gal4-VP64 can minimize the unexpected activation of non-target genes and crosstalk between synNotch signaling and native signaling. One of the disadvantages of the synNotch system is immune rejection due to non-human-derived components; however, this can be avoided using fully humanized synNotch synthetic intramembrane proteolysis receptors (SNIPRs), which are expected to enter clinical use shortly (113).

## Discussion

Various secretory co-factor–expressing CAR T cells are under development to improve their anti-tumor efficacy and safety. It is difficult to directly compare the ability of secretory co-factors to enhance the efficacy of CAR T cells against solid tumors because no ideal animal models of human solid tumors have yet been established. However, ICBs, IL-12, IL-15, and IL-18 have the advantage of existing clinical data for their recombinant administration in patients with advanced or metastatic solid tumors (69, 71, 76, 77). Indeed, several clinical trials of PD-1– or PD-L1–secreting CAR T-cell therapies are ongoing in China (**Table 2**), and we anticipate that at least one of these cytokines will be tested as a co-factor for CAR T cells in the near future. Due to the abundance of different types of solid tumors, multiple CAR T-cell types should be developed to allow clinicians to make the best therapeutic choice for each patient. For instance, IFN-γ enhances ICAM-1–mediated CAR T-cell cytotoxicity against various types of solid tumor but not the leukemia, lymphoma, or myeloma CDX models (114), although this different IFN-γ sensitivities are unclear in clinical level. In addition, multiple cytokines and ICBs can be loaded onto a CAR construct to exert additive or synergistic effects against more tumor types. IL-7 and CCL19/CCL21 combinations, which are intended to recruit and maintain nonengineered immune cells, are leading the race for optimal co-factor combinations for CAR T-cell therapies (32–34); however, various co-factor combinations must be tested after single co-factor CAR T-cell clinical trials.

In terms of genetic engineering, wherein the co-factor gene is added to a CAR-coding construct, “next-generation” CAR T cells are expected to have equivalent or even better efficacy than promising combination therapies involving the same factor without additional administration. To maximize clinical outcomes, the automated regulation of the amount and timing of co-factor expression could be the next development focus. For instance, IL-6 and IL-1 blockades are promising co-factors to prevent CRS; however, their constitutive expression might be problematic due to unexpected immunosuppressive effects. “CAR T-cell activation–dependent” or “tumor site–specific” expression machineries could solve this issue. Although weak NFAT promoter activity is the biggest concern for activation-dependent machinery, the modification of the NFAT promoter and the newly verified NR4A promoter could enhance maximum co-factor production. SynNotch is a synthetic biology solution for antigen-dependent high co-factor expression, whereas hypoxia-inducible promoters can achieve site-specific expression to maximize co-factor efficacy. In addition, genome-editing techniques could produce various types of endogenous promoters that could be used simultaneously to control multiple co-factors.

To regulate the spatial range of effects more strictly, synthetic chimera forms of cytokines have also been reported. Membrane-bound cytokines are chimera forms fused to a transmembrane domain or a cell surface receptor. Expressing the membrane-bound cytokines is intended to enhance the potency of engineered cells themself and minimize the effect on surrounding cells, which is supposed to work as a conditioned culture even *in vivo* (115–117). Therefore, this strategy could strongly support recently spotlighted fast CAR T-cell manufacturing procedures that expect *in vivo* expansion of CAR T cells instead of conventional *ex vivo* expansion (118, 119). Another synthetic chimera form of cytokines is a target-tethered cytokine composed of a cytokine and an antibody fragment or a specific binding domain (68, 120). TME-specific accumulation or immune cell–specific tethering can enhance the anti-tumor efficacy of IL-12 while limiting systemic toxicities. Similar to BiTE, zipFv-zipCAR, and CD19-BP (in **Section 4**), this approach could be applied to CAR T-cell therapy as a secretory co-factor that plays an immunomodulatory role in synthetic cell-cell communication.

In summary, synthetic biology approaches could expand the T-cell engineering tool kit and enable CAR T cells to be programmed with more complex functionality. Beyond cancer therapy, future secretory co-factors could enable synthetic communication between engineered cells and nonengineered cells or among engineered cells to build a synthetic immune cell consortium. Altogether, combining CAR T cells with secretory co-factors will lead to next-generation CAR T-cell therapies against broader types of cancers and provide advanced tools for more complicated synthetic immunotherapies.

## Author contributions

AO, YI, TK, SH, and SA prepared the manuscript. All authors contributed to the article and approved the submitted version.

## Funding

AO was supported by Leading Initiative for Excellent Young Researchers grant from Japan Society for the Promotion of Science.

## Conflict of interest

AO, YI, TK, SH, and SA are currently full-time employees at Hitachi, Ltd.

## Publisher’s note

All claims expressed in this article are solely those of the authors and do not necessarily represent those of their affiliated organizations, or those of the publisher, the editors and the reviewers. Any product that may be evaluated in this article, or claim that may be made by its manufacturer, is not guaranteed or endorsed by the publisher.
